# Causal association of NAFLD with osteoporosis, fracture and falling risk: a bidirectional Mendelian randomization study

**DOI:** 10.3389/fendo.2023.1215790

**Published:** 2023-08-09

**Authors:** Aiyong Cui, Peilun Xiao, Zhiqiang Fan, Jinlai Lei, Shuang Han, Danlong Zhang, Xing Wei, Pengfei Wang, Yan Zhuang

**Affiliations:** ^1^ Department of Orthopaedics, Honghui Hospital, Xi’an Jiao Tong University, Xi’an, China; ^2^ Department of Orthopaedics, The Fifth Affiliated Hospital of Sun Yat-Sen University, Zhuhai, Guangdong, China

**Keywords:** NAFLD, osteoporosis, fracture, Mendelian randomization, genome-wide association study

## Abstract

**Introduction:**

The causal association between non-alcoholic fatty liver disease (NAFLD) and osteoporosis remains controversial in previous epidemiological studies. We employed a bidirectional two-sample Mendelian analysis to explore the causal relationship between NAFLD and osteoporosis.

**Method:**

The NAFLD instrumental variables (IVs) were obtained from a large Genome-wide association study (GWAS) meta-analysis dataset of European descent. Two-sample Mendelian randomization (MR) analyses were used to estimate the causal effect of NAFLD on osteoporosis, fracture, and fall. Reverse Mendelian randomization analysis was conducted to estimate the causal effect of osteoporosis on NAFLD. The inverse-variance weighted (IVW) method was the primary analysis in this analysis. We used the MR-Egger method to determine horizontal pleiotropic. The heterogeneity effect of IVs was detected by MR-Egger and IVW analyses.

**Results:**

Five SNPs (rs2980854, rs429358, rs1040196, rs738409, and rs5764430) were chosen as IVs for NAFLD. In forward MR analysis, the IVW-random effect indicated the causal effect of NAFLD on osteoporosis (OR= 1.0021, 95% CI: 1.0006-1.0037, *P*= 0.007) but not on fracture (OR= 1.0016, 95% CI: 0.998-1.0053, *P*= 0.389) and fall (OR= 0.9912, 95% CI: 0.9412-1.0440, *P*= 0.740). Furthermore, the reverse Mendelian randomization did not support a causal effect of osteoporosis on NAFLD (OR= 1.0002, 95% CI: 0.9997-1.0007, *P*= 0.231). No horizontal pleiotropic was detected in all MR analyses.

**Conclusions:**

The results of this study indicate a causal association between NAFLD and osteoporosis. NAFLD patients have a higher risk of osteoporosis but not fracture and falling risk. In addition, our results do not support a causal effect of osteoporosis on NAFLD.

## Introduction

Osteoporosis (OP) is a bone disorder featured by low bone mass density (BMD) and impaired microarchitecture, leading to a higher fragility fracture risk for the old (1). In a meta-analysis, the global prevalence of osteoporosis in people aged 50-59, 60-69, and 70-79 was 11.4%, 24.8, and 37.6%, respectively (2). In 2013, twenty-two million females and 5.5 million males in European Union (EU) countries suffered from osteoporosis, leading to 3.5 million fragility fractures annually (3). Osteoporosis is a multifactorial disease involving clinical, nutritional, behavioral, medical, and genetic factors in their occurrence (4). Imbalance in bone formation and resorption due to various reasons, such as reduced estrogen levels in postmenopausal women or advanced age, will lead to osteoporosis (5). Previously, gender, age, family osteoporosis history, alcohol consumption, smoking, hormone use, some diseases, and others were considered possible risk factors for osteoporosis (6, 7).

Non-alcoholic fatty liver disease (NAFLD) has become the most common chronic liver disease, threatening about 25% population worldwide (8). With the epidemic of the western diet, sedentary lifestyle, and obesity, the prevalence of NAFLD in the general population has been increasing in recent years (9). NAFLD spectrum includes non-alcoholic fatty liver (NAFL), non-alcoholic steatohepatitis (NASH), and NASH-related end-stage liver disease, such as cirrhosis. Insulin resistance, type 2 diabetes (T2D), hyperlipidemia, hypertension, and metabolic syndrome may play an essential role in the development of NAFLD (10). In previous studies, a large number of studies have demonstrated the possible role of NAFLD in the development of osteoporosis. Studies showed that the prevalence of osteoporosis in NAFLD patients is higher than in patients without NAFLD (11). NAFLD may affect osteoporosis or osteoporotic fractures in several ways, including the changes in bone metabolic transforming factors, vitamin D levels, the chronic inflammatory state of the liver, degree of hepatic fibrosis, and disturbances in lipid metabolism, and others. However, previous observational studies investigating the association between NAFLD and osteoporosis have consistently yielded inconsistent results. Some studies have shown that NAFLD is associated with lower BMD or higher fracture risk in adolescents or adults (12–14), while others have shown no significant correlation or even opposite results (15–17). These observational studies have obvious limitations like unmeasured or imprecisely measured confounders such as gender, age, menstrual status, and others, which inevitably lead to some biases.

Mendelian randomization (MR) is a novel genetic variation method to assess the causal relationship between exposure and outcome (18). It could avoid confounding factors and infer causality since the alleles of exposure genetic variants are randomly assigned (19). Thus, we employed an MR analysis to explore the causal effect of NAFLD on osteoporosis, fracture, and falling risk. A reverse MR analysis was also used to explore the causal effect of osteoporosis on NAFLD.

## Materials and methods

### Study design

**Figure 1:**
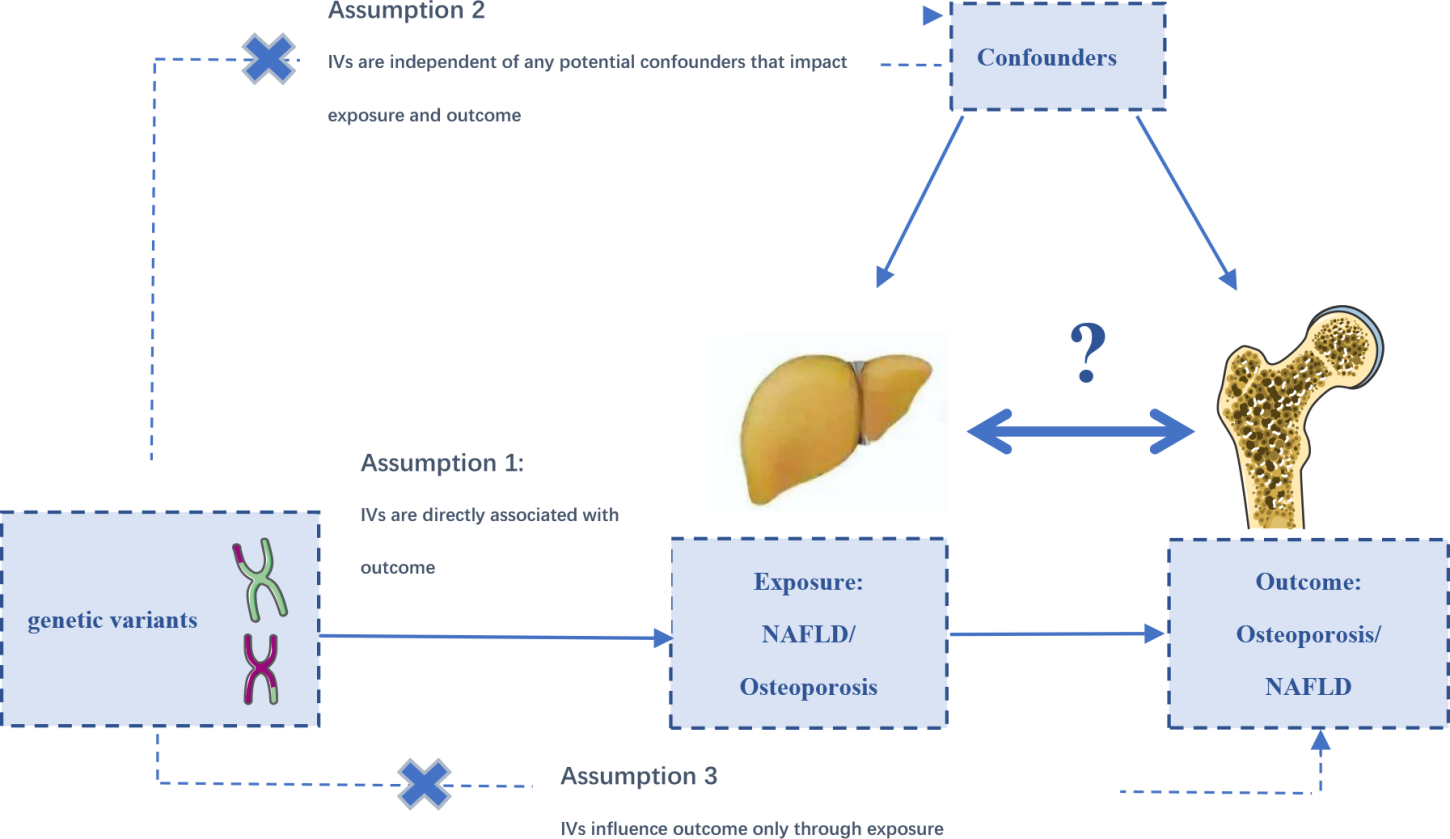
The designs and assumptions for the bidirectional Mendelian randomization study of the causal association of NAFLD and osteoporosis, fracture, and falling risk.

The MR analyses conform to three assumptions: 1. Selected SNPs should be strongly correlated with exposure. 2. Selected SNPs are not related to the outcome through confounders. 3. Selected SNPs are supposed to affect outcomes *via* exposure, but not the direct association (**Figure 1**). All information in this study was obtained from public databases or existing publications, which did not need additional ethical approval.

### Data sources for NAFLD

The sources of NAFLD were obtained from large GWASs by Ghodsian et al. (20). The GWASs meta-analysis included summary statistics from the FinnGen cohorts eMERGE, the latest GWAS conducted by the Estonian Biobank (4,119 cases and 190,120 controls), and a new NAFLD GWAS from the UK Biobank (2,558 cases and 395,241 controls), with a total of 8,434 NAFLD cases and 770,180 controls. In this GWAS meta-analysis, the associations were conducted by adjusting for gender, age, and ten main ancestry-based principal components and genotyping (20). The electronic health record documented NAFLD with participants of European ancestry with hepatic steatosis, NASH, or liver fibrosis (LF). Finally, five SNPs (rs2980854, rs429358, rs10401969, rs738409, rs5764430) closely associated with NAFLD (p< 5×10^-08^) without linkage were chosen as alternative instruments for NAFLD (R^2^<0.001, window size =10,000 kb).

### Data sources for osteoporosis, fractures, and fall

Summary-level genetic data on osteoporosis were collected from the UK Biobank (UK Biobank: 20002#1309), including 7,547 osteoporosis samples and 455,386 control samples. The genetic associations with fracture risk were revealed in a large GWAS meta-analysis combining 23andMe cohorts and the UK Biobank, with a total of 1.2 million subjects (21). The fracture diagnosis was defined as a break in bone continuity at any site in the past five years, except for fractures at hands, feet, skull, or pathological fractures due to malignancy, infection, and others (21). GWAS data for falling susceptibility were collected from a UK Biobank study, involving 89,076 fall cases and 362,103 controls (22). A “fall” is defined when the subject gives an affirmative answer to the following question: “Have you fallen in the last year?

### Statistical analysis

The bidirectional MR approach was conducted to estimate the causality between NAFLD and osteoporosis, fracture, and fall. Relevant SNPs were selected when genome-wide significance was less than p< 5×10^-8^. SNPs were excluded when detecting linkage disequilibrium (LD) (R^2^ > 0.001 or clump distance< 10,000 kb). Five methods were employed to examine the causal association, including inverse-variance weighted (IVW), MR-Egger, weighted mode, weighted median, and simple model, among which IVW was the primary method (23). Random-effects IVW was performed to assess the genetic predictions between them. The MR-Egger method was used to test its horizontal pleiotropic. Outlier SNPs were detected by MR-PRESSO packages and then deleted. Cochran’s Q statistic was applied to examine the heterogeneity of individual SNPs in IVW and MR-Egger tests. We additionally performed sensitivity analysis by removing single SNP one by one. To evaluate the weak instrument bias, we calculated the F statistics using the formula $\text{F}=\left(\frac{N-K-1}{K}\right)\left(\frac{R^{2}}{1-R^{2}}\right)$ , where N is the sample size, K is the number of IVs, and *R* ^2^ is the proportion of the variability of the exposure explained by IVs. All data were analyzed by package “TwoSampleMR” of the R language.

## Results

### Genetical prediction of NAFLD on osteoporosis

**Figure 2:**
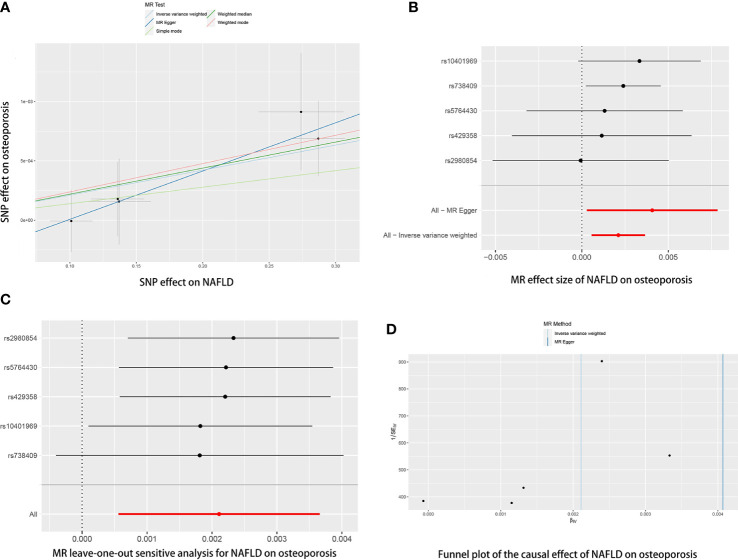
The causal effects of NAFLD on osteoporosis in different MR methods. **(A)** Scatter plot. The slopes of each line represent the causal association for each method. **(B)** Forrest plot. **(C)** Leave-one-out analysis. **(D)** Funnel plot.

Five SNPs (rs2980854 in/near TRIB1, rs429358 in/near APOE, rs10401969 in/near SUGP1, rs738409 in/near PNPLA3, rs5764430 in/near SAMM50) were chosen as IVs for NAFLD (**Supplementary Table 1**). The SNP was larger than the empirical threshold 10 of the F statistics, indicating a weak bias of the IVs. The causal association of NAFLD variants with osteoporosis was shown in **Table 1** and **Figure 2**. The IVW results found a positive effect of NAFLD on osteoporosis (OR= 1.0021, 95% CI: 1.0006-1.0037, *P*= 0.007). The consistent result was also found in MR Egger (OR= 1.0041, 95% CI: 1.0003-1.0087, *P*= 0.012), weighted median (OR= 1.0022, 95% CI: 1.0004-1.0040, *P*= 0.015), and weighted mode (OR= 1.0023, 95% CI: 1.0002-1.0045, *P*= 0.024). The Cochran’s Q statistic of MR-Egger (*P*= 0.972) and IVW methods (*P*= 0.831) indicated no significant heterogeneity between IVs. No horizontal pleiotropy was detected in MR–Egger intercept (*P*= 0.346). In the leave-one-out sensitive analyses, we found rs738409 strongly drove the overall effect of NAFLD on osteoporosis (**Figure 2C**). Furthermore, the MR-PRESSO test did not identify any potential SNP outliers.

**Table 1:** Causal effect of NAFLD on osteoporosis using genetic variant randomization in different MR methods.

Exposure	Outcome	Method	No. of SNP	OR (95% CI)	95% CI	*P*
NAFLD	Osteoporosis	IVW	5	1.0021	1.0006-1.0037	0.007
		MR Egger	5	1.0041	1.0003-1.0087	0.012
		Weighted median	5	1.0022	1.0004-1.0040	0.015
		Simple mode	5	1.0014	0.9987-1.0041	0.375
		Weighted mode	5	1.0023	1.0002-1.0045	0.024
MR Egger: Cochran’s Q= 0.234, *P*= 0.972						
IVW: Cochran’s Q= 1.476, *P*= 0.831						
MR-Egger intercept= -0.0004, *P*= 0.346						
MR-PRESSO global test= 0.868						

No outlier was observed in the MR-PRESSO analysis in MR analysis in NAFLD and osteoporosis. CI, confidence interval; MR, Mendelian randomization; IVW, inverse-variance weighted; NAFLD, non-alcoholic fatty liver disease.

### Genetical prediction of NAFLD on fracture risk

**Figure 3:**
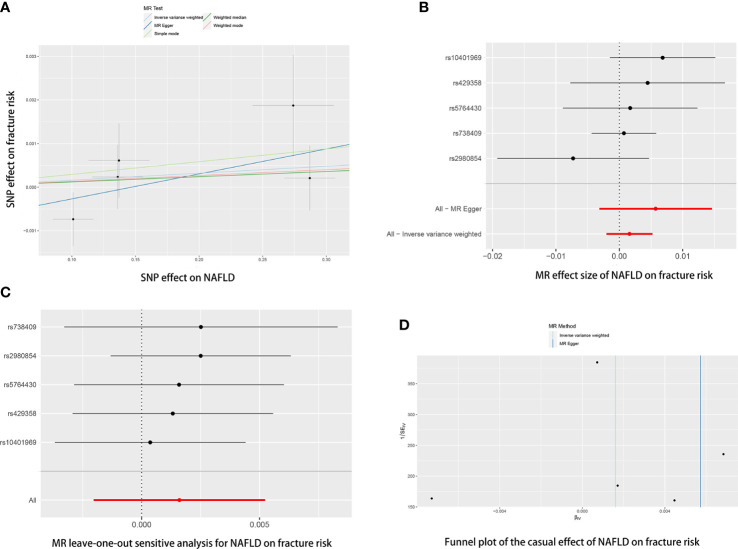
The causal effects of NAFLD on fracture risk in different MR methods. **(A)** Scatter plot. The slopes of each line represent the causal association for each method. **(B)** Forrest plot. **(C)** Leave-one-out analysis. **(D)** Funnel plot.

The causal association of NAFLD variants with fracture was shown in **Table 2** and **Figure 3**. MR analysis by the IVW method showed that there was no causal effect of NAFLD on fracture (OR= 1.0016, 95% CI: 0.998-1.0053, *P*= 0.389). The consistent result was also found in MR Egger (OR= 1.0057, 95% CI: 0.9969-1.0147, *P*= 0.295), weighted median (OR= 1.0012, 95% CI: 0.9970-1.0055, *P*= 0.581), simple mode (OR= 1.0029, 95% CI: 0.9961-1.0098, *P*= 0.448) and weighted mode (OR= 1.0013, 95% CI: 0.9963-1.0064, *P*= 0.632). The Cochran’s Q statistic of MR-Egger (*P*= 0.396) and IVW methods (*P*= 0.411) indicated no significant heterogeneity between IVs. No horizontal pleiotropy was detected in MR–Egger intercept (*P*= 0.391). In addition, the result remains no significant after excluding a single SNP (**Figure 3C**). MR-PRESSO test did not identify any potential SNP outliers.

**Table 2:** Causal effect of NAFLD on fracture risk using genetic variant randomization in different MR methods.

Exposure	Outcome	Method	No. of SNP	OR (95% CI)	95% CI	*P*
NAFLD	Fracture	IVW	5	1.0016	0.998-1.0053	0.389
		MR Egger	5	1.0057	0.9969-1.0147	0.295
		Weighted median	5	1.0012	0.9970-1.0055	0.581
		Simple mode	5	1.0029	0.9961-1.0098	0.448
		Weighted mode	5	1.0013	0.9963-1.0064	0.632
MR Egger: Cochran’s Q= 2.967, *P*= 0.396						
IVW: Cochran’s Q= 3.965, P= 0.411						
MR-Egger intercept= -0.0008, *P*= 0.391						
MR-PRESSO global test= 0.517						

No outlier was observed in the MR-PRESSO analysis in MR analysis in NAFLD and fractures. CI, confidence interval; MR, Mendelian randomization; IVW, inverse-variance weighted; NAFLD, non-alcoholic fatty liver disease.

### Genetical prediction of NAFLD on falling risk

**Figure 4:**
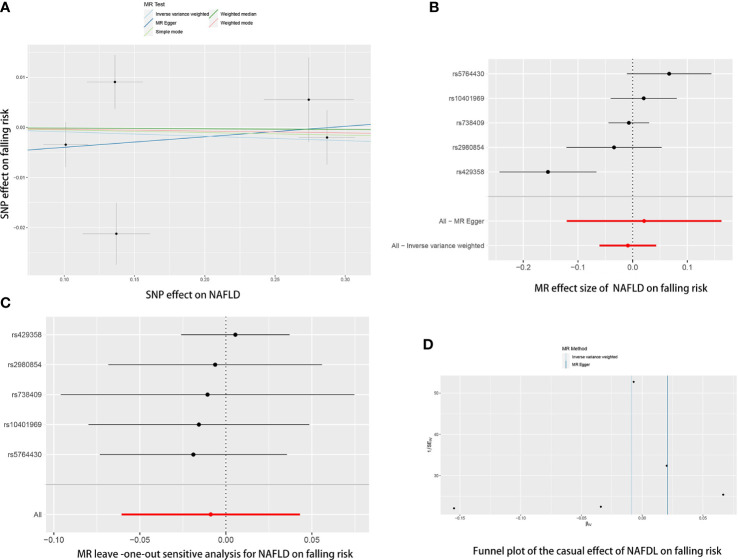
The causal effects of NAFLD on falling risk in different MR methods. **(A)** Scatter plot. The slopes of each line represent the causal association for each method. **(B)** Forrest plot. **(C)** Leave-one-out analysis. **(D)** Funnel plot.

The causal association of NAFLD variants with fracture was shown in **Table 3** and **Figure 4**. MR analysis by the IVW method showed that there was no causal effect of NAFLD on falling risk (OR= 0.9912, 95% CI: 0.9412-1.0440, *P*= 0.740). The consistent result was also found in MR Egger (OR= 1.0211, 95% CI: 0.8865-1.1763, *P*= 0.791), weighted median (OR= 0.9987, 95% CI: 0.9681-1.0301, *P*= 0.932), simple mode (OR= 0.9949, 95% CI: 0.9521-1.0396, *P*= 0.831) and weighted mode (OR= 0.9964, 95% CI: 0.9613-1.0327, *P*= 0.852). The Cochran’s Q statistic of MR-Egger (*P*= 0.002) and IVW methods (*P*= 0.004) indicated significant heterogeneity between IVs, suggesting the use of the random effect model of IVW. No horizontal pleiotropy was detected in MR–Egger intercept (*P*= 0.682). The result remains no significant in leave-one-out sensitive analyses (**Figure 4C**). MR-PRESSO did not identify any potential SNP outliers.

**Table 3:** Causal effect of NAFLD on falling risk using genetic variant randomization in different MR methods.

Exposure	Outcome	Method	No. of SNP	OR (95% CI)	95% CI	*P*
NAFLD	Falling risk	IVW	5	0.9912	0.9412-1.0440	0.740
		MR Egger	5	1.0211	0.8865-1.1763	0.791
		Weighted median	5	0.9987	0.9681-1.0301	0.932
		Simple mode	5	0.9949	0.9521-1.0396	0.831
		Weighted mode	5	0.9964	0.9613-1.0327	0.852
MR Egger: Cochran’s Q= 14.341, *P*= 0.002						
IVW: Cochran’s Q= 15.315, *P*= 0.004						
MR-Egger intercept= -0.006, *P*= 0.682						
MR-PRESSO global test= 0.756						

No outlier was observed in the MR-PRESSO analysis in MR analysis in NAFLD and fractures. CI, confidence interval; MR, Mendelian randomization; IVW, inverse-variance weighted; NAFLD, non-alcoholic fatty liver disease.

### Reverse Mendelian randomization analysis

**Figure 5:**
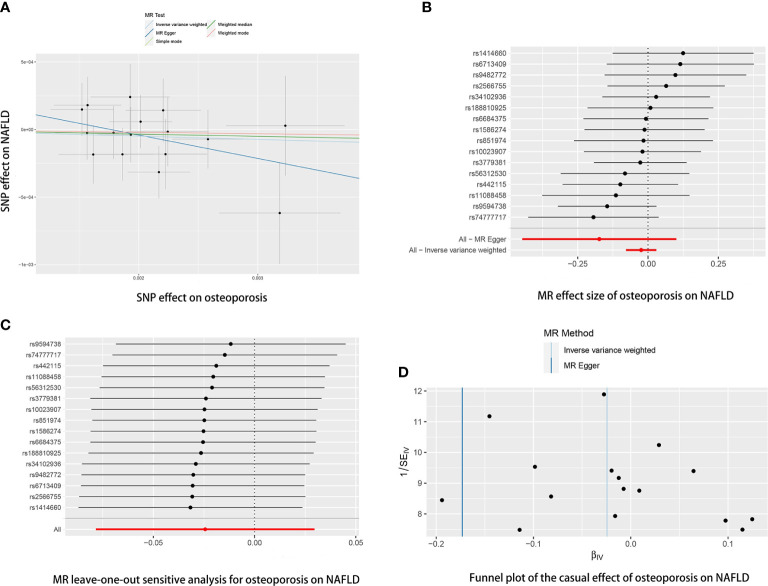
The causal effects of osteoporosis on NAFLD in different MR methods. **(A)** Scatter plot. The slopes of each line represent the causal association for each method. **(B)** Forrest plot. **(C)** Leave-one-out analysis. **(D)** Funnel plot.

Reverse MR analyses were then conducted to assess the causal effect of osteoporosis on NAFLD. Sixteen SNPs without linkage were chosen as the IVs for osteoporosis (**Supplementary Table 2**). Our study found no causal effect of osteoporosis on NAFLD (OR= 0.9759, 95% CI: 0.9246-1.3000, *P*= 0.375) (**Table 4** and **Figure 5**). The F-statistic for IVs was 60.2 > 10. The consistent results were also found in other MR methods. The Cochran’s Q statistic of MR-Egger (*P*= 0.869) and IVW methods (*P*= 0.847) indicated no significant heterogeneity between IVs. The result remains no significant in leave-one-out sensitive analyses (**Figure 5C**). No horizontal pleiotropy was detected in MR–Egger intercept (*P*= 0.296). MR-PRESSO also did not identify any potential SNP outliers.

**Table 4:** Causal effect of osteoporosis on NAFLD using genetic variant randomization in different MR methods.

Exposure	Outcome	Method	No. of SNP	OR (95% CI)	95% CI	*P*
Osteoporosis	NAFLD	IVW	16	0.9759	0.9246-1.3000	0.375
		MR Egger	16	0.8410	0.6393-1.1062	0.235
		Weighted median	16	0.9834	0.9143-1.0578	0.653
		Simple mode	16	0.9898	0.8753-1.1192	0.872
		Weighted mode	16	0.9897	0.8818-1.1101	0.864
MR Egger: Cochran’s Q=8.365, *P*= 0.869						
IVW: Cochran’s Q= 9.542, P= 0.847						
MR-Egger intercept= 0.0003, *P*= 0.296						
MR-PRESSO global test= 0.856						

No outlier was observed in the MR-PRESSO analysis in MR analysis in osteoporosis and NAFLD. CI, confidence interval; MR, Mendelian randomization; IVW, inverse-variance weighted; NAFLD, non-alcoholic fatty liver disease.

## Discussion

Although much work has explored the potential link between NAFLD and osteoporosis, the results are hardly convincing because of the nature of observational studies that cannot overcome the confounding factors. To our knowledge, this is the first MR study to assess the causal association between NAFLD and osteoporosis. Our studies indicated that genetic prediction of NAFLD was associated with a higher risk of osteoporosis. However, our findings did not support a causal association of NAFLD variants with fractures and falling risk. Moreover, the reverse MR analysis showed no genetic prediction of NAFLD on osteoporosis. In this study, five variations (rs2980854, rs429358, rs10401969, rs738409, rs5764430) at the APOE, SUGP1, TRIB1, SAMM50, and PNPLA3 loci linked to NAFLD was chosen for analysis. There are two known susceptibility loci for NAFLD (SAMM50, PNPLA3) and three potentially new candidate genetic regions for a clinical NAFLD diagnosis (APOE, SUGP1, TRIB1) (20).

Previously, several potential mechanisms behind NAFLD in osteoporosis have been widely discussed, including vitamin D deficiency, increased cytokines from the inflamed liver, limited physical activity, and others. In NAFLD, chronic production of pro-inflammatory cytokines like tumor necrosis factor (TNF)-a, interleukin (IL)-1, IL-6, and IL-7. TNF-a may be an important cytokine that mediates bone loss in NAFLD patients. Several studies have reported increased levels of circulating TNF-a in patients with NAFLD (24, 25). Studies showed that increased levels of TNF-α lead to increased osteoclast production and inhibit osteoblast activation (26). In addition, studies have indicated that serum TNF-a level was negatively associated with vitamin D levels (27). Osteocalcin (OCN) is a non-collagen protein expressed by osteoblasts, works as a marker of bone formation, and involves calcium homeostasis. Fernández-Real et al. (28) reported that serum OCN level was negatively associated with the blood markers of liver diseases, such as alanine transaminase (ALT) and aspartate transaminase (AST). In a cohort of 47 subjects, Szalay et al. (29) reported a decreased serum OCN level in NAFLD patients (29). At the same time, OCN may play a role in postmenopausal osteoporosis (30). In a recent study by Xu et al. (31), decreased OCN was associated with bone turn markers and osteopenia, and osteoporosis. Thus, decreased OCN in NAFLD patients could be a potential factor leading to osteoporosis. Osteopontin (OPN) is another multi-functional protein in our body, which is regarded as another factor that may mediate low BMD in NAFLD. Syn et al. (32) demonstrated that OPN was overexpressed in NAFLD patients. They found that the level of OPN is also linked with fibrosis progression in NASH in transgenic mice. On the other hand, higher OPN is also known as a risk of osteoporosis (33, 34). In an animal study, Chiang et al. showed that OPN-deficient mice could resist ovariectomy-induced osteoporosis (33). Another human study by Chang et al. (34) showed the same conclusion. They found a higher serum OPN levels (>14.7 ng/ml) are an essential risk factor for menopausal osteoporosis. Studies have shown that OPN may regulate the function of osteoblasts and osteoclasts by inhibiting the growth of mineral crystals (35). Vitamin D deficiency in NAFLD patients may provide another explanation for osteoporosis tendency. Targher et al. (36) found that NAFLD patients have a lower serum vitamin D level than the control group, and the level of vitamin D decrease was closely related to the histological severity of hepatic steatosis. In a recent study, Ciardullo et al. (37) found an inverse relationship between vitamin D status and NAFLD and fibrosis. The evidence suggests that vitamin D deficiency may play a role in NAFLD occurrence and progression. Furthermore, insufficient physical activity is also an essential factor in developing NAFLD. Physical activity is considered an important treatment to prevent NAFLD and reduce fat accumulation in the liver. At the same time, much clinical evidence has shown that limited physical inactivity is essential to osteoporosis in the elderly. Thus, insufficient physical activity could be an important lifestyle link to NAFLD with osteoporosis.

All in all, much evidence supports the tendency of osteoporosis in NAFLD patients *in vivo* and *in vitro* experiments. However, the previous results in clinical observational studies on their association were always controversial. In a retrospective cross-sectional study including 3,739 postmenopausal women, Lee et al. (38) indicated a significant negative association between NAFLD and BMD after adjusting potential confounders. A recent meta-analysis encompassing 7 observational studies showed a similar conclusion (12). In the analysis, Pan et al. showed that NAFLD patients have a higher risk of osteoporosis (OR = 1.33, 95%CI:1.24-1.44) and osteoporotic fractures (OR = 1.57, 95%CI:1.08-2.29) (12). However, after parameter adjustment, the significant association was only found in men but not in women (12). In a recent NHANES study, Xie et al. (39) found a negative association between NAFLD and osteoporosis. However, the association correlation becomes insignificant after adjusting for a large range of covariates, including gender, age, race, poverty income ratio (PIR), body mass index (BMI), and others. In another NAHENS study of 1784 subjects older than 50, Ciardullo et al. (40) shown the same findings. After adjusting for confounders, they found no association between NAFLD and femoral BMD and osteoporosis. These conflicting findings may result from the confounding factor in observational studies. For example, obesity may be an important mediator of the relationship between NAFLD and osteoporosis. In an early study by Li et al. (41), they found a positive association between NAFLD and osteoporosis in multivariable-adjusted models. However, after additionally adjusting visceral adipose tissue and BMI, they found NAFLD was negatively associated with vertebral BMD.

These epidemiological studies are prone to generate biased results because of the residual confounding and reverse causation (42). We first use MR analysis to reveal a causal association between NAFLD and osteoporosis risk. However, although the IVW method showed that NAFLD may increase fracture risk, the results were not statistically significant. Our results do not support that NAFLD increases the risk of fracture. Fracture is influenced by many factors. Our results show that NAFLD only slightly increased osteoporosis risk, so NAFLD may not cause a significantly increased fracture risk. In addition, our results also do not support that NAFLD increases the falling risk. Our study has several advantages. First, MR analysis was designed to reduce confounding and reverse causality using a genetic variation, as SNPs are randomly assigned at conception. Second, we used a large sample of the GWAS meta-analysis dataset (8,434 NAFLD cases and 770,180 controls) to conduct this MR analysis. Large F-statistic values indicated five IVs were closely related to NAFLD. Third, we also performed a reverse MR analysis to explore the causal effect of osteoporosis on NAFLD, which facilitates our better understanding of the interconnection between these two diseases. However, some potential limitations could not be avoided. First, some heterogeneity is found between NAFLD and fracture, which could be led by the choice of some instrumental variables. Second, this MR study reveals the potential causal effect of NAFLD on osteoporosis, but no causal association is observed between NAFLD and fracture and falling risk. The inconsistent result remains elusive and needs further investigation. Third, due to the limitations of GWAS, we did not perform MR analysis by age and sex. Fourth, another thing worth mentioning is that although we found a higher osteoporosis risk in NAFLD patients, this increase was small, and the OR was quite low. So, the conclusion should be interpreted more cautiously. Fifth, these findings should be interpreted cautiously, as the study sample was from a European population. Whether the results can be generalized to other populations will need to be further investigated.

## Conclusions

The results of this study indicate a causal association between NAFLD and osteoporosis. NAFLD patients have a higher risk of osteoporosis but not fractures and falling risk. In addition, our results do not support a causal effect of osteoporosis on NAFLD.

## Data availability statement

The original contributions presented in the study are included in the article/**Supplementary Material**. Further inquiries can be directed to the corresponding authors.

## Ethics statement

Ethical review and approval was not required for the study on human participants in accordance with the local legislation and institutional requirements. Written informed consent for participation was not required for this study in accordance with the national legislation and the institutional requirements.

## Author contributions

Conceptualization: AC, PX, PW. Data curation: ZF, XW, and YZ. Formal analysis, XW and YZ. Investigation: AC, PX, and ZF. Methodology: AC, PX, ZF, JL, SH, and DZ. Project administration: AC, PX, and PW. Software: AC, PX, and ZF. Visualization: HW and YZ. Writing – original draft: AC, PX, ZF, DZ, XW, PW, and YZ. Writing – review & editing, AC, PX, and ZF. All authors contributed to the article and approved the submitted version.
