# Supplementary material for: Causal association of NAFLD with osteoporosis, fracture and falling risk: a bidirectional Mendelian randomization study
Source: Front Endocrinol (Lausanne). 2023 Aug 9;14:1215790. doi: 10.3389/fendo.2023.1215790 (PMC10446969; doi:10.3389/fendo.2023.1215790)
Supplement: Supplementary file 1 [file Table_1.docx]

**Supplementary Table 1**: Associations of genetic instruments for NAFLD with osteoporosis.

|  |  |  |  |  |  |  | SNP-exposure (NAFLD) | | |  | SNP-outcome (Osteoporosis) | | |
| --- | --- | --- | --- | --- | --- | --- | --- | --- | --- | --- | --- | --- | --- |
| SNP | Gene | Sample size | EA | OA | EAF |  | Beta | SE | P |  | Beta | SE | P |
| rs10401969 | TRIB1 | 462933 | C | T | 0.07 |  | 0.274 | 0.032 | 7.58E-18 |  | 0.00091 | 0.00050 | 0.06500 |
| rs2980854 | APOE | 462933 | C | T | 0.55 |  | 0.101 | 0.016 | 6.22E-10 |  | -0.00001 | 0.00026 | 0.98000 |
| rs429358 | SUGP1 | 462933 | T | C | 0.84 |  | 0.137 | 0.024 | 1.14E-08 |  | 0.00016 | 0.00036 | 0.66000 |
| rs5764430 | PNPLA3 | 462933 | C | A | 0.89 |  | 0.136 | 0.02 | 7.19E-12 |  | 0.00018 | 0.00031 | 0.57000 |
| rs738409 | SAMM50 | 462933 | G | C | 0.23 |  | 0.287 | 0.02 | 1.23E-47 |  | 0.00069 | 0.00032 | 0.03000 |

***P* value < 5×10^-8^ for reporting genome-wide significance. Abbreviations: EA, effect allele. OA, other allele, EAF effect allele frequency. MR, Mendelian randomization. SE, standard error; SNP, single nucleotide polymorphism. NAFLD, non-alcoholic fatty liver disease.**
