# Supplementary material for: Causal association of NAFLD with osteoporosis, fracture and falling risk: a bidirectional Mendelian randomization study
Source: Front Endocrinol (Lausanne). 2023 Aug 9;14:1215790. doi: 10.3389/fendo.2023.1215790 (PMC10446969; doi:10.3389/fendo.2023.1215790)
Supplement: Supplementary file 2 [file Table_2.docx]

**Supplementary Table 2**: Associations of genetic instruments for osteoporosis with NAFLD.

|  |  |  |  |  |  | SNP-exposure (Osteoporosis) | | | SNP-outcome (NAFLD) | | |
| --- | --- | --- | --- | --- | --- | --- | --- | --- | --- | --- | --- |
| SNP | Nearby Gene | Sample size | EA | OA | EAF | Beta | SE | P | Beta | SE | P |
| rs10023907 | MEPE |  | T | C | 0.00028 | 0.00193 | 0.00028 | 3.10027E-12 | 0.00355 | 0.01742 | 0.83840 |
| rs11088458 | LINC01700 |  | G | A | 0.00029 | 0.00162 | 0.00029 | 2.99999E-09 | -0.00536 | 0.01763 | 0.76120 |
| rs1414660 | FMN2 |  | T | C | 0.00033 | -0.00193 | 0.00033 | 6.80002E-09 | 0.00425 | 0.02169 | 0.84480 |
| rs1586274 | NA | 778,614 | G | A | 0.00026 | 0.00179 | 0.00026 | 1.10002E-11 | 0.01422 | 0.01625 | 0.38140 |
| rs188810925 | RP11-209M4.1 | 778,614 | A | G | 0.00050 | -0.00323 | 0.00050 | 8.49963E-11 | -0.04274 | 0.02876 | 0.13730 |
| rs2566755 | WLS | 778,614 | C | T | 0.00032 | -0.00221 | 0.00032 | 3.29989E-12 | -0.00856 | 0.02085 | 0.68140 |
| rs34102936 | STARD3NL | 778,614 | A | G | 0.00027 | -0.00202 | 0.00027 | 3.29989E-14 | 0.02077 | 0.01651 | 0.20850 |
| rs3779381 | WNT16 | 778,614 | G | A | 0.00030 | -0.00258 | 0.00030 | 6.59933E-18 | 0.02614 | 0.01857 | 0.15920 |
| rs442115 | RP11-944L7.4 | 778,614 | G | T | 0.00026 | -0.00186 | 0.00026 | 1.69981E-12 | 0.00312 | 0.01633 | 0.84840 |
| rs56312530 | LRP5 | 778,614 | A | G | 0.00035 | 0.00223 | 0.00035 | 2.30001E-10 | -0.01886 | 0.02423 | 0.43620 |
| rs6684375 | LRP5 | 778,614 | T | C | 0.00034 | -0.00224 | 0.00034 | 6.70039E-11 | -0.01020 | 0.02351 | 0.66430 |
| rs6713409 | RP11-415K20.2 | 778,614 | A | T | 0.00028 | -0.00157 | 0.00028 | 2.80001E-08 | -0.01860 | 0.01743 | 0.28580 |
| rs74777717 | RP5-877J2.1 | 778,614 | G | C | 0.00051 | 0.00318 | 0.00051 | 3.89996E-10 | -0.07779 | 0.03448 | 0.02408 |
| rs851974 | P11-15G8.1 | 778,614 | A | G | 0.00027 | 0.00157 | 0.00027 | 4.09996E-09 | -0.01899 | 0.01643 | 0.24780 |
| rs9482772 | RSPO3 | 778,614 | C | T | 0.00026 | -0.00152 | 0.00026 | 7.90005E-09 | 0.04278 | 0.01630 | 0.00868 |
| rs9594738 | RP11-413N19.2 | 778,614 | T | C | 0.00026 | 0.00217 | 0.00026 | 1.20005E-16 | -0.00425 | 0.01622 | 0.79320 |

***P* value < 5×10^-8^ for reporting genome-wide significance. Abbreviations: EA, effect allele. OA, other allele, EAF effect allele frequency. MR, Mendelian randomization. SE, standard error; SNP, single nucleotide polymorphism. NAFLD, non-alcoholic fatty liver disease.**
